# MicroRNAs and suicidality: a systematic review and bioinformatic evaluation

**DOI:** 10.3389/fpsyt.2026.1723187

**Published:** 2026-01-30

**Authors:** Mahdi Malekpour, Mohammadreza Akbari, Mobin Fallah Tafti, Kimia Falamarzi, Fahimeh Golabi, Mohammad Javad Entezari Meybodi, Kamyab Shahrivar, Niayesh Ghasemi, Farzad Midjani, Nemat Jaafari, Murray J. Cairns

**Affiliations:** 1 Research Center for Psychiatry and Behavioral Sciences, Shiraz University of Medical Sciences, Shiraz, Iran; 2 Systems Medicine Research Core, Shiraz University of Medical Sciences, Shiraz, Iran; 3 Nephro-Urology Research Center, Shiraz University of Medical Sciences, Shiraz, Iran; 4 Research Committee, Shiraz University of Medical Sciences, Shiraz, Iran; 5 Université de Poitiers, Unité de Recherche Clinique du Centre Hospitalier Henri Laborit, Équipe de Recherche CoCliCo CNRS UMR 7295, Poitiers, France; 6 School of Biomedical Sciences and Pharmacy, The University of Newcastle, Callaghan, NSW, Australia; 7 Precision Medicine Research Program, Hunter Medical Research Institute, New Lambton Heights, NSW, Australia

**Keywords:** bioinformatics, microRNA, miRNA, suicidality, suicide

## Abstract

**Introduction:**

Suicide is a leading global cause of mortality (~800,000 deaths annually) driven by complex biological and environmental determinants; although microRNAs (miRNAs) regulate gene expression implicated in psychiatric disorders, their contributions to suicidality-related phenotypes remain incompletely defined.

**Methods:**

We searched Web of Science, PubMed, Scopus, Embase, and Ovid through July 14, 2025, for human case–control studies comparing individuals with suicidality-related phenotypes to non-suicidal controls. Risk of bias was assessed with the Newcastle–Ottawa Scale. Differentially expressed miRNAs were compiled and analyzed to identify brain-specific gene targets, followed by pathway and disease enrichment.

**Results:**

Of 1,437 records screened, 13 studies met inclusion criteria, encompassing 285 suicidal participants and 291 controls. Across studies, 43 unique miRNAs showed significant differential expression between cases and controls. Three miRNAs—miR-30a, miR-30e, and miR-218—were consistently dysregulated across brain samples from individuals who died by suicide. Bioinformatic analyses indicated that these miRNAs converge on brain-expressed targets and processes relevant to psychiatric biology. Enrichment highlighted pathways involved in transcriptional regulation, forkhead box O (FoxO) signaling, Ras-associated protein-1 (Rap1) signaling, long-term depression, and dopaminergic synapse function.

**Conclusion:**

miR-30a, miR-30e, and miR-218 emerge as recurrently altered miRNAs in suicide and may serve as mechanistic mediators and candidate biomarkers. Mapping their brain-specific targets and enriched pathways suggests actionable avenues for risk stratification and therapeutic development.

**Systematic Review Registration:**

https://www.crd.york.ac.uk/prospero/, identifier PROSPERO CRD42024582398.

## Introduction

Suicide is a serious global public health problem that deeply affects individuals and communities worldwide, with an estimated 800,000 people dying by suicide each year (1). The causes of suicide are complex and multifactorial, involving both genetic and environmental factors (2). Psychiatric disorders are significant risk factors across all suicidality-related phenotypes, with approximately 90% of cases involving individuals who had a diagnosable psychiatric condition at the time of death (3). Genome-wide association studies (GWAS) have identified numerous loci associated with suicidality-related phenotypes, with the strongest genetic correlations observed between suicidal ideation or suicide attempts and major depressive disorder (MDD) (4).

microRNA (miRNA) are small noncoding RNA molecules that function as potent regulators of gene expression and play important roles in brain development, synaptic function and stress responses (5, 6). miRNA can be investigated not only in specific neural cells but also in a variety of body fluids, including whole blood, plasma, serum, cerebrospinal fluid (CSF), and saliva. This extensive accessibility and functionality render miRNA as promising candidates for diagnostic and therapeutic applications across a wide range of physiological and pathological conditions (7, 8).

Interestingly, approximately 50% of all known miRNA have been identified in the human brain and are associated with target genes that play key roles in neuronal function, including synaptogenesis and plasticity (9). As our understanding of impact of miRNA on gene regulation expands, their potential significance in the context of neurodevelopmental syndromes and neuropsychiatric disorders has become increasingly apparent (10, 11).

Recent studies have begun to explore the role of miRNA across different suicidality-related phenotypes. For instance, alterations in the expression of certain miRNA have been observed in postmortem human brain studies from individuals who died by suicide (12–14). These findings suggest that miRNA could potentially serve as biomarkers of suicidality, providing new insights into the molecular mechanisms underlying suicidal ideation, suicide attempts, and death by suicide (15).

Bioinformatics tools have proven effective in identifying brain-specific targets of dysregulated miRNAs, mapping affected pathways, and clarified mechanisms underlying suicidality, informing biomarkers and therapeutic targets (16–18).

Given the global public health burden of suicidality, understanding the underlying molecular mechanisms that contribute to suicidal ideation, suicide attempts, and death by suicide is crucial for developing effective preventive and therapeutic strategies. In this study, we aim to review existing research on the role of miRNA in suicidality, with the goal of understanding the molecular mechanisms that influence suicide-related phenotypes through these miRNA. To do this, we will use a variety of bioinformatics tools.

## Materials and methods

To elucidate the molecular pathways involved in suicide, we initiated our research by conducting a systematic review. The primary objective of this review was to identify miRNA that would subsequently be subjected to our bioinformatics analysis. The protocol for the systematic review phase of this study has been registered on PROSPERO (CRD42024582398).

### Study selection criteria

In this systematic review, we exclusively considered studies with case-control designs on humans. The cases were required to include individuals who had died by suicide, engaged in suicide attempts or non-suicidal self-injury (NSSI), or experienced suicidal ideation, irrespective of whether these phenotypes were reported subjectively or objectively. Suicidal ideation refers to thoughts of ending one’s life without enacted behavior; suicide attempt denotes non-fatal self-injurious behavior with intent to die; death by suicide refers to a fatal outcome of self-injurious behavior with suicidal intent; and NSSI is defined as deliberate self-injury without intent to die (19). The term “suicidality” is used as an umbrella construct encompassing these related but distinct phenotypes.

In contrast, the control group was required to consist solely of non-suicidal individuals. Our outcome measure was a direct comparison of microRNA expression levels between the case and controls. Notably, the presence of comorbidities among individuals included in a study was not a determining factor for inclusion or exclusion.

Furthermore, we excluded studies that did not report any significant dysregulation in miRNA levels associated with suicide. Additionally, we limited our analysis to studies published in the English language, and we excluded review articles, conference summaries, and letters, as well as studies employing non–case–control designs.

### Database search strategies

A comprehensive and systematic literature search was conducted in Web of Science, PubMed, Scopus, Embase, and Ovid databases without initial date restrictions, up to and including July 14, 2025. The search queries used for each database are detailed in **Supplementary Material 1**. The initial search yielded a total of 1,437 references.

### Study selection process

All retrieved references were managed using EndNote X9 software. Duplicate references were initially removed. Two reviewers (MJEM and MFT) independently assessed the titles and abstracts of these articles. Subsequently, the full texts of selected articles were evaluated by two authors (MFT and KF) based on the inclusion and exclusion criteria. Any discrepancies in the selection process were resolved through discussion or by involving a third reviewer (MM) to reach consensus. The systematic review adhered to the Preferred Reporting Items for Systematic Reviews and Meta-Analyses (PRISMA) guidelines (**Supplementary Material 2**) (20).

### Data collection

From each included study’s full text and **Supplementary Data**, two reviewers (MFT and MM) independently extracted the following data items: first author, year of publication, study country, tissue types, sample sizes, age and gender distribution of cases and controls, underlying comorbidities among all individuals, phenotype of exposure in case individuals (death by suicide vs. NSSI vs. suicidal ideation), type of miRNA expression assay, lists of upregulated and downregulated miRNA with statistically significant expression changes, and criteria used for determining differential expression. Extracted data were compared, and any disagreements were resolved through discussion or by involving a third reviewer (MRA) to achieve consensus.

### Risk of bias assessment

Two authors (MRA and KF) independently appraised the included studies using the Newcastle-Ottawa Scale (NOS), a quality assessment tool for case-control studies. The NOS employs a “star system” based on three major criteria: selection of the study groups (0–4 stars), comparability of the groups through control of the most relevant factors (0–2 stars), and ascertainment of the outcome of interest (0–3 stars). A total score of three or less indicated poor quality, 4–6 indicated moderate quality, and 7–9 indicated high quality. Any disagreements regarding the risk of bias in specific studies were resolved through discussion, with the involvement of a third reviewer (MM) if necessary.

### miRNA target prediction and brain-specific filtering

To elucidate miRNA effects on brain function in suicidality, first the genes affected by each miRNA predicted using the miRNA data integration portal (mirDIP). mirDIP integrates microRNA-human gene interactions from more than 30 databases. It provides an integrated likelihood score for each microRNA-human gene interaction (21, 22). In the subsequent step, for finding brain specific targets, only genes with detectable brain expression filtered using The Human Protein Atlas (23). In the next step, to identify connections between the targets of miRNAs, the MolBioTools list comparator was used to illustrate a Venn diagram (molbiotools.com/listcompare.php).

### Enrichment analysis

For finding the molecular processes affected by each miRNA in the brain, Enrichr knowledge graph (Enrichr-KG) database was used. Enrichr-KG is an enrichment analysis tool that integrate enrichment analysis between multiple databases (24). Individual and shared brain-specific targets were imported to Enrichr-KG for analysis based on Kyoto Encyclopedia of Genes and Genomes (KEGG) (25–27) and Gene Onthology (GO) (28, 29) libraries. Additionally, to identify diseases associated with our gene sets, an enrichment analysis was also performed using the DisGeNET database (30).

## Results

### Study selection process

**Figure 1:**
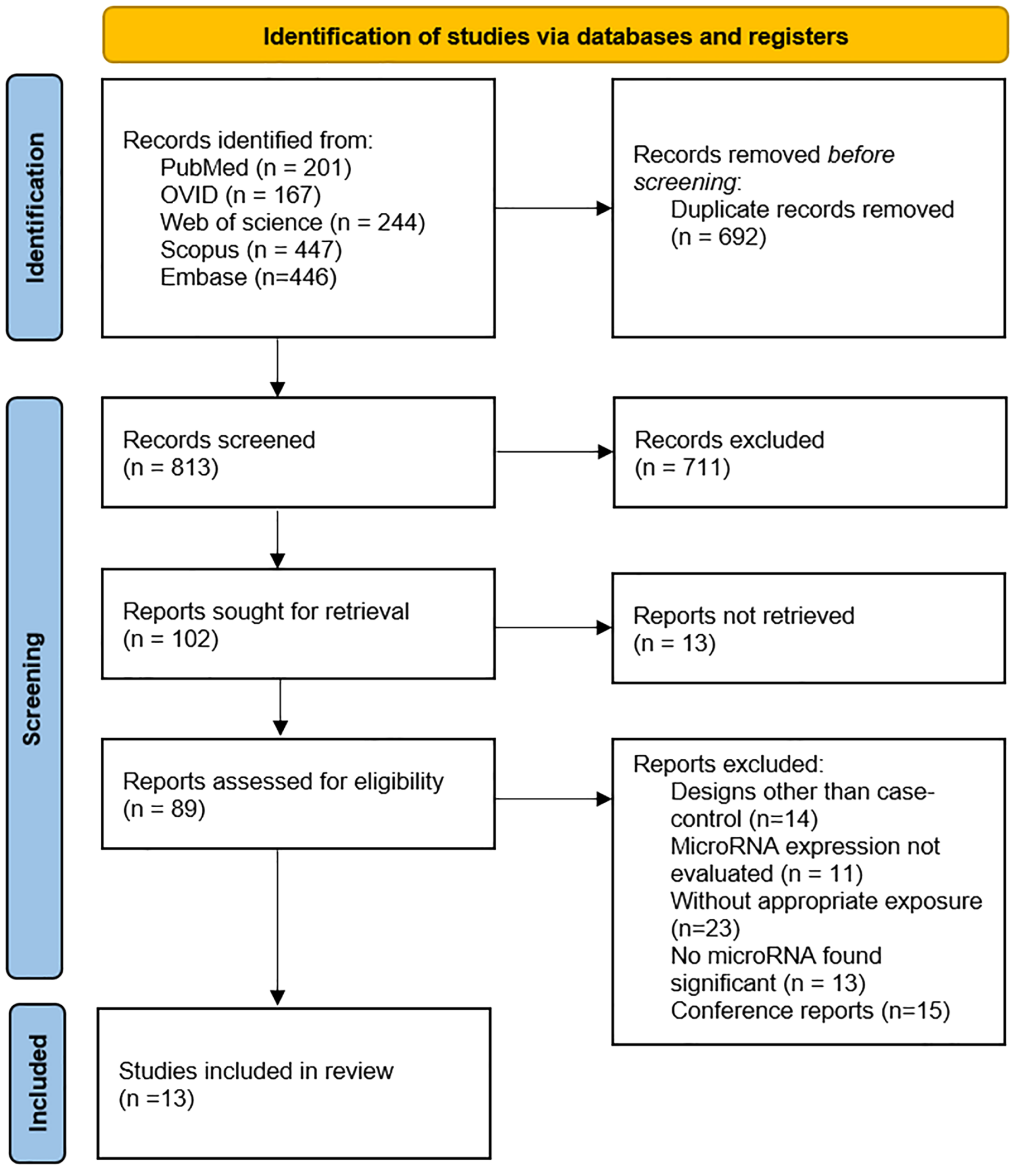
Flow diagram of included studies.

The database search across Web of Science, PubMed, Scopus, Embase and OVID databases yielded a total of 1,437 studies. After removing duplicates, 803 articles were subjected to title and abstract screening, of which 98 articles were selected for full-text evaluation. Ultimately, 13 studies met the eligibility criteria and were included in this systematic review. The PRISMA flowchart depicting the study selection process is presented in **Figure 1**.

### Characteristics of included studies

The 13 included studies, published between 2012 and 2023, examined a total of 285 individuals with suicidality-related phenotypes and 291 controls (detailed in **Supplementary Material 3**).

Four studies enrolled only male participants (12–14, 31). Ten studies analyzed postmortem brain tissues (12–14, 32–40), one used blood serum (41), one examined peripheral blood leukocytes (42), and one assessed CSF (31).

Among the brain tissue studies:

- Six studies compared MDD patients who died by suicide with non-psychiatric controls (12, 14, 32–34, 36).
- Two studies assessed individuals who died by suicide, with or without major psychiatric disorders compared with non-psychiatric controls (13, 37).

In the two blood-based studies, all subjects had MDD (41, 42). The CSF study compared extracellular vesicles from individuals who died by suicide with those from individuals who died from sudden cardiac death (31).

**Figure 2:**
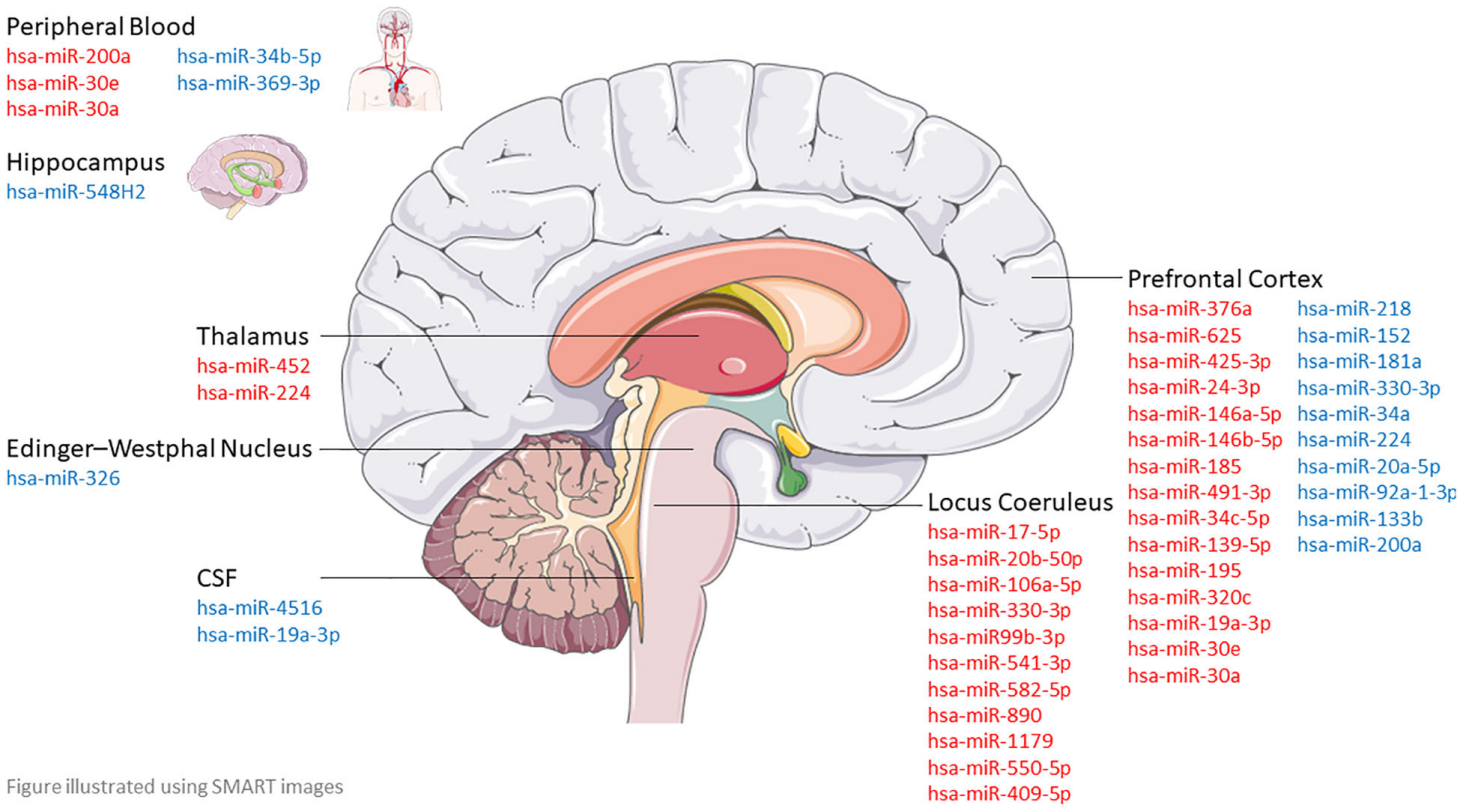
Dysregulated microRNA across different brain regions based on previous studies, with red indicating upregulated miRNA and blue indicating downregulated miRNA.

Across all studies, 43 unique miRNAs showed significantly differential expression between cases and controls (**Supplementary Material 3**). **Figure 2** illustrates dysregulated miRNAs across brain regions. miR-200a, miR-224, miR-19a, and miR-330-3p were dysregulated in separate studies but showed inconsistent directionality (upregulated in one, downregulated in another; **Table 1**). miR-30a, miR-30e, and miR-218 were consistently dysregulated in brain samples from individuals who died by suicide. These were prioritized for subsequent bioinformatics analysis (**Table 2**). miR-30a and miR-30e were upregulated (36, 41), miR-218 was downregulated in two different cohorts from Quebec Corner’s office and Douglas-Bell Canada brain bank (34).

**Table 1:** Inconsistently dysregulated miRNA related to suicide.

miRNA	Status	Country	Case group	Control group	Number (Case/Control)	Case male: female	Control male: female	Fold change	References
miR-200a	Down-regulated	Germany	MDD and death by suicide	non-psychiatric	30 (14/16)	8M: 6F	10M: 6F	N/A	(36)
	Up-regulated	Germany	MDD and - acute suicide risk or suicide attempt	MDD	50 (19/31)	6 M: 13 F	10 M: 21 F	N/A	(41)
miR-224	Down-regulated	USA	MDD, BD, and schizophrenia who were death by suicide cases	MDD, BD, schizophrenia, and non-psychiatric controls without suicidality	56 (16/40)	N/A	N/A	-0.57	(38)
	Up-regulated	Canada	Death by suicide cases	non-psychiatric	27 (20/7)	11 M: 9 F	4 M: 3 F	2.1	(37)
miR-330-3p	Down-regulated	USA	MDD, bipolar, and schizophrenia who were death by suicide cases	MDD, bipolar, schizophrenia, and non-psychiatric controls without suicidality	56 (16/40)	N/A	N/A	-0.53	(38)
	Up-regulated	USA	MDD and death by suicide cases	Non-psychiatric	20 (9/11)	6 M: 3 F	9 M:2 F	1.4	(33)
miR-19a-3p	Down-regulated	Slovenia	Death by suicide cases	Sudden cardiac arrest	20 M	20 M	N/A	N/A	(31)
	Up-regulated	USA	Death by suicide cases	non-psychiatric	59 (43/16)	36 M: 7 F	16 M	5	(35)

miRNA, microRNA; MDD, major depressive disorder; BD, bipolar disorder; N/A, not available; M, male; F, female.

**Table 2:** Consistently dysregulated miRNA related to suicide, these three miRNA were subjected to bioinformatics analysis.

miRNA	Country	Case group	Control group	Number (Case/Control)	Case male: female	Control male: female	Fold change	References
Upregulated miRNA								
mir-30a and mir-30e	Germany	MDD and - death by suicide	Non-psychiatric	30 (14/16)	8M: 6F	10M: 6F	N/A	(36)
	Germany	MDD and - acute suicide risk or suicide attempt	MDD	50 (19/31)	6 M: 13 F	10 M: 21 F	N/A	(41)
Downregulated miRNA								
mir-218	Canada	MDD and death by suicide	Non-psychiatric	59 (24/35) in original cohort	22 M: 2 F	31 M:4 F	-2.71	(34)
				23 (11/12) in validation cohort	11 M	12 M	-2.3	

miRNA, microRNA; MDD, Major Depressive Disorder; M, male; F, female; N/A, not available.

**Supplementary Material 4** details the risk of bias assessment. Of the 13 studies one study was rated as having a moderate risk of bias; however, its exclusion did not alter the miRNAs selected for bioinformatics analysis.

### Brain-specific gene targets of each miRNA

**Figure 3:**
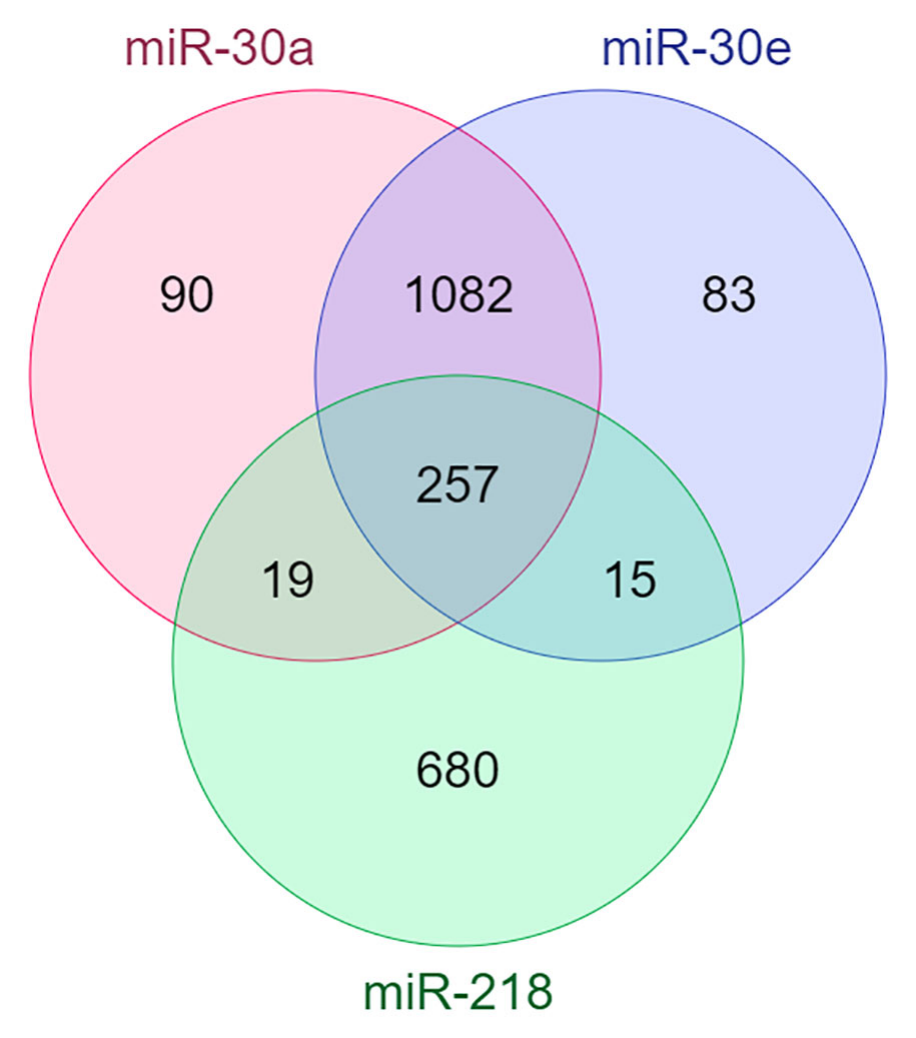
The Venn diagram showing the number of shared targets between miRNA.

According to mirDIP database, miR-30a targets 1646 genes, miR-30e targets 1639 and miR-218 targets 1003 genes with high probability. After restricting predicted gene targets to those expressed in the brain, miR-30a was found to target 1,448 genes, miR-30e to target 1,437 genes, and miR-218 to target 971 genes. In total, 257 target genes were shared among these three miRNAs. The Venn diagram of connection between targets of these three miRNA showed in **Figure 3**. (**Supplementary Material 5**).

### Pathways associated with gene targets

**Figure 4:**
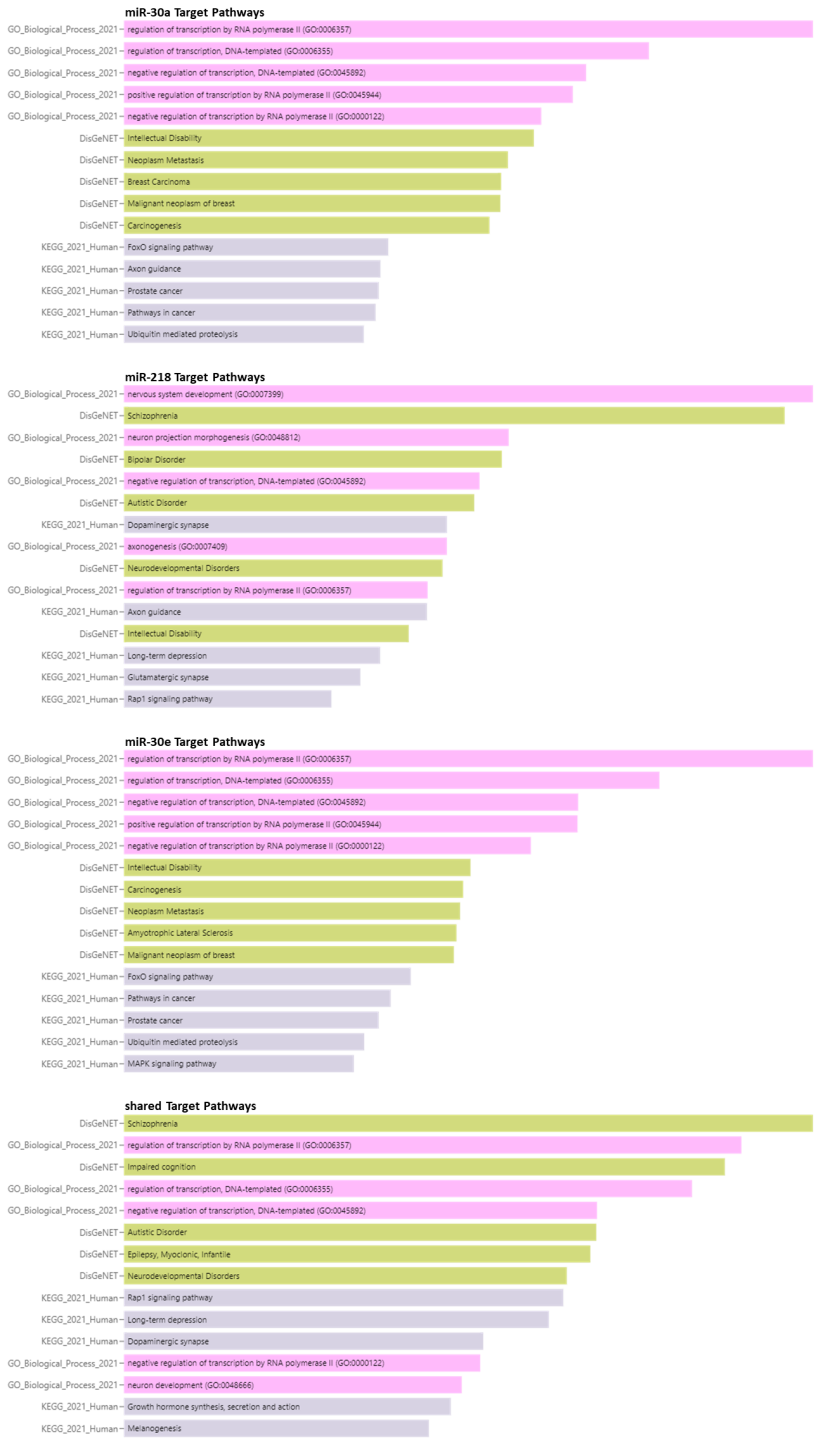
Enriched pathways associated with miRNA targets. Pink rows are GO Biological pathways, golden rows are DisGeNET associated pathways and golden rows are diseases associated with miRNA target genes based on KEGG.

The enrichment analysis identified several enriched pathways, as illustrated in **Figure 4**. As anticipated, the pathways enriched for miR-30a and miR-30e were largely overlapping, reflecting their shared gene targets. Both of these miRNA targeted the regulation of DNA transcription, forkhead box O (FoxO) signaling, cancer pathways, and ubiquitin-mediated proteolysis. Moreover, the targets of miR-30a and miR-30e were associated with various diseases such as intellectual disability, carcinogenesis, and metastasis. Interestingly, miR-30a targets were also linked to axon guidance, while miR-30e targets were associated with Amyotrophic Lateral Sclerosis (ALS) and mitogen−activated protein kinase (MAPK) signaling.

In contrast, miR-218 primarily targeted pathways related to nervous system development, neuron projection morphogenesis, axogenesis, dopaminergic and glutaminergic synapse, axon guidance, long-term depression, and Ras-associated protein-1 (Rap1) signaling. The targets of miR-218 were also linked to psychiatric diseases such as schizophrenia (SCZ), bipolar disorder (BD), and neurodevelopmental disorders including autism.

Furthermore, the shared targets of these three miRNA were associated with the regulation of transcription, Rap1 signaling, long-term depression, dopaminergic synapse, and growth hormone synthesis, secretion, and action. These shared targets were also linked to schizophrenia, impaired cognition, autistic disorder, myoclonic infantile epilepsy, and other neurodevelopmental disorders. Collectively, these findings provide insight into the potential biological roles of these miRNAs in suicidality-related phenotypes and related neuropsychiatric conditions.

## Discussion

The biological basis of suicidal behavior is incompletely defined (43), and available treatments for suicidality show limited efficacy (44). This systematic review identified 13 human case-control studies evaluating miRNA expression in relation to suicidality. Across 285 suicidal participants and 291 controls, 43 unique miRNAs were reported as differentially expressed. Only three miRNAs were consistently dysregulated across independent datasets. miR-30a and miR-30e were upregulated in both suicidal-ideation cohorts and death by suicide cases (36, 41), while miR-218 was downregulated in death by suicide cases. In contrast, miR-200a showed tissue- and stage-specific patterns, being upregulated in blood from suicidal-ideation participants (41) but downregulated in brain tissue from death by suicide cases (36). These findings warrant confirmation in larger, prospective studies, but may support future risk stratification and early identification of individuals at elevated risk of suicide.

Prior works connects miR-30a with stress exposure and neuroplasticity, including associations with childhood trauma in depression (45). miR-30a’s upregulation in the prefrontal cortex (PFC) can lead to increased alcohol consumption and decreased expression of brain-derived neurotrophic factor (BDNF), a factor linked to depressive symptoms, BD, and SCZ (46–49). Increased level of miR-30e also identified in the PFC of death by suicide cases (50). Moreover, miR-30e has also been implicated in MDD and SCZ. miRNA-30e levels reduce following pharmacological treatment in SCZ, aligning with symptom improvement (51). miR-30e influences depression-like symptoms induced by chronic stress through its effects on neurogenesis and neuroplasticity in the hippocampus (34).

miR-218 is predominantly expressed in CNS and has established roles in stress susceptibility and depression-related biology (52–55). Experimental and postmortem studies have associated reduced miR-218 with stress vulnerability and depressive symptoms (34, 56–58). In the present analysis, miR-218 targets were enriched for synaptic and neurodevelopmental pathways, including dopaminergic and glutamatergic synapse function and long-term depression. These findings align with earlier studies linking dopaminergic and glutamatergic mechanisms to miR-218 and suicidal behavior, as well as reports of rapid anti-suicidal effects of ketamine through modulation of glutamatergic synapses (59–65). Furthermore, a large GWAS on U.S. veterans with a history of suicide attempts revealed an overrepresentation of dopaminergic and glutamatergic pathways (61). Additionally, miR-218 is involved in harmful brain changes in MDD patients through epigenetic irregularities, with alterations in its expression in the hippocampus linked to changes in the activity of the Hypothalamic–Pituitary–Adrenal (HPA) axis (56, 66, 67).

FoxO signaling was the top enriched KEGG pathway among miR-30a and miR-30e target genes. FoxO signaling is an important pathway that regulates the stress resistance, neural development and neurogenesis (68) (69). FoxO signaling plays an important role in the pathophysiology of SCZ, MDD, and anxiety (70). However, to the best of our knowledge, the association between FoxO signaling and suicide has not been described before. It appears that chronic stress can induce HPA-axis activation, decreased BDNF level, and decreased serotonin and norepinephrine in the brain, which can result in dysregulation of FoxO signaling. This can cause cellular atrophy and decreased neurogenesis, leading to depressive disorders (71).

Ubiquitin-Mediated Proteolysis (UMP) was another enriched pathway among miR-30a and miR-30e targets. Multi-omics studies have also implicated this pathway in SCZ (72), BD and psychosis (73), drug addiction (74), and depression (75). Although specific mechanisms remain uncertain, proposed effects include altered protein remodeling, synaptic function, and neural plasticity, which are relevant to cognition and affect regulation (74, 76, 77).

Shared-target enrichment across miR-30a, miR-30e, and miR-218 highlighted Rap1 signaling, long-term depression, dopaminergic synapse, and growth hormone–related pathways. Rap1 signaling has been implicated in stress-related cortical dysfunction and has also been reported as altered in brains of death by suicide cases (78) (79). Preclinical works also supports a role for Rap1 in synaptic plasticity and fear learning within the amygdala (80). Also, stress can led to the overexpression of Rap1, resulting in cognitive impairments (78). These findings indicate that despite the limited understanding of the role of Rap1 signaling in psychiatric disorders, it could potentially be a significant target for the treatment of these diseases.

Growth hormone signaling has clinical links with mood and behavioral phenotypes in deficiency states, and earlier work reported associations between growth hormone response and suicidal behavior in depression (81). Previous research indicates that growth hormone deficiency (GHD) leads to psychological complications, and growth hormone replacement significantly improves mood and quality of life in patients (81). GHD has a correlation with generalized anxiety disorder (GAD) and social anxiety disorder (SAD) (82). Furthermore, the function of the growth hormone, by altering the sensitivity of dopaminergic synapses, may potentially contribute to suicide attempts in depressed patients (83).

One of the other pathways enriched based on miR-218 and miR-30a targets is axon guidance. The importance of these two miRNA in axon development and re-wiring has been confirmed in previous laboratory studies (57, 84, 85). Epigenetic alterations, like miRNA dysregulations, can change neural circuits which had been previously observed in an epigenetic study on depressed death by suicide cases (86).

Several neuropsychiatric disorders, including intellectual disability, ALS, SCZ, BD, ASD neurodevelopmental disorders, long-term depression, and impaired cognition, have been enriched based on the targets of the miRNA. The association of many of these diseases, such as SCZ, BD, and MDD, with suicide has been previously established (87, 88). On the other hand, the key role of miR-218, miR-30a and miR-30e as susceptibility regulatory factor to stress-induced diseases, especially depression, has been proven before (34) (45, 89) has been previously identified. The enrichment of these neuropsychiatric diseases with the targets of our miRNA underscores the significant importance of these miRNA in psychiatric diseases related to suicide and possibly suicide itself.

### Limitations

This study has some limitations. Studies evaluated microRNA profiles from various samples and brain regions, leading to inconsistencies. There is also substantial heterogeneity among control groups, with some studies including depressive or schizophrenic controls and others including controls with no psychiatric history. This heterogeneity, combined with the fact that the consistent dysregulation of certain miRNAs (e.g., miR−30a, miR−30e, and miR−218) was reported in only a small number of studies, highlights the confounding effects of comorbidities such as MDD or BD in suicide research. As a result, these miRNAs should be considered potential mediators and biomarkers with caution, and further validation studies with better-matched controls and fewer comorbid conditions are needed to assess their specificity and utility for risk stratification or clinical application. In addition, some studies included only male participants, making it unclear whether specific miRNA dysregulations are gender-specific. Finally, research specifically addressing the effects of miRNA in suicide pathways remains limited, which constrained the scope of our comparisons and required reliance on evidence from other psychiatric disorders.

## Conclusion

This study highlights the crucial role of miRNA, specifically miR-30a, miR-30e, and miR-218, in the pathophysiology of suicide. The enrichment of targets of these miRNAs revealed several pathways, including FoxO signaling, UMP, and Rap1 signaling. According to these findings miRNA found in this study can significantly influence key cellular functions and signaling pathways associated with psychiatric disorders. Furthermore, the association of these miRNA with disorders such as intellectual disability, ALS, SCZ, BD, ASD, and neurodevelopmental disorders reflects their importance in psychiatric diseases related to suicide. The study provides promising directions for future research in suicide prevention, with these miRNA offering potential as biomarkers or targets for therapeutic development.

## Data Availability

The datasets presented in this study can be found in online repositories. The names of the repository/repositories and accession number(s) can be found in the article/**Supplementary Material**.
