# Supplementary material for: MicroRNAs and suicidality: a systematic review and bioinformatic evaluation
Source: Front Psychiatry. 2026 Jan 30;17:1723187. doi: 10.3389/fpsyt.2026.1723187 (PMC12900758; doi:10.3389/fpsyt.2026.1723187)
Supplement: Supplementary file 1 [file Table1.docx]

Search strings

Date of search: Last updated on 14 July 2025

Pubmed:

("Suicide"[Mesh] OR Suicid*[Title/Abstract] OR "suicidal ideation"[Title/Abstract] OR "suicide attempt"[Title/Abstract] OR "non-suicidal self-injury"[Title/Abstract] OR "self-cutting"[Title/Abstract] OR self-injur*[Title/Abstract] OR self-harm*[Title/Abstract] OR Parasuicid*[Title/Abstract]) AND ("MicroRNAs"[Mesh] OR MicroRNA OR miRNA* OR "pri-miRNA" OR "Micro RNA" OR miRNome OR miR OR microRNA* OR "small RNA" OR "stRNA" OR "Small Temporal RNA" OR "non-coding RNA" OR "RNAi" OR "RNA interference")

OVID:

1. Books from Ovid to Full Text - University Health Science Foundation - ASCOFAME
2. All Ovid Magazines (Sumbs, Abstract, Underwritten Magazines)
3. Magazines underwritten to Full Text by the University Health Sciences Foundation - ASCOFAME
4. EBM Reviews - ACP Journal Club 1991 to June 2025
5. EBM Reviews - Cochrane Central Register of Controlled Trials June 2025
6. EBM Reviews - Cochrane Database of Systematic Reviews 2005 to July 9, 2025
7. EBM Reviews - Cochrane Clinical Answers June 2025
8. Ovid Emcare 1995 to 2025 Week 27
9. Ovid MEDLINE ALL(R) / PubMed(R) / 1946 to Present

(suicide or suicid* or "suicidal ideation" or "suicide attempt" or "non-suicidal self-injury" or "self-cutting" or "self-injur*" or "self-harm*" or Parasuicid*).ab,ti,sh,kw. and (microRNA or miRNA or "pri-miRNA" or "Micro RNA" or miRNome or microRNA* or "small RNA" or "non-coding RNA" or mir or "stRNA" or "Small Temporal RNA" OR "RNAi" OR "RNA interference").fx,kf,sh.

Scopus:

( TITLE-ABS-KEY ( suicide OR suicid* OR "suicidal ideation" OR "suicide attempt" OR "non-suicidal self-injury" OR "self-cutting" OR self-injur* OR self-harm* OR parasuicid* ) AND TITLE-ABS-KEY ( "MicroRNAs" OR microrna OR mirna* OR "pri-miRNA" OR "Micro RNA" OR mirnome OR "stRNA" OR "Small Temporal RNA" OR miR OR microrna* OR "small RNA" OR "non-coding RNA" OR "RNAi" OR "RNA interference" ) )

Embase:

('suicide'/exp OR 'suicid*':ti,ab,kw OR 'suicidal ideation':ti,ab,kw OR 'suicide attempt':ti,ab,kw OR 'non-suicidal self-injury':ti,ab,kw OR 'self-cutting':ti,ab,kw OR 'self-injur*':ti,ab,kw OR 'self-harm*':ti,ab,kw OR 'parasuicid*':ti,ab,kw) AND ('microRNA'/exp OR 'microrna' OR 'mirna*' OR 'pri-mirna' OR 'micro rna' OR 'mirnome' OR 'mir' OR 'microrna*' OR 'small rna' OR 'strna' OR 'small temporal rna' OR 'non-coding rna' OR 'rnai' OR 'rna interference')

Web of Science Core Collection:

(TS=(Suicide OR Suicid* OR "suicidal ideation" OR "suicide attempt" OR "non-suicidal self-injury" OR "self-cutting" OR "self-injur*" OR "self-harm*" OR "Parasuicid*") AND TS=(MicroRNAs OR MicroRNA OR miRNA* OR “pri-miRNA” OR Micro RNA OR miRNome OR miR OR microRNA* OR “small RNA” OR “small non-coding RNA” or "stRNA" or "Small Temporal RNA" OR "RNAi" OR "RNA interference"))

Hits per database:

Pubmed : 201

OVID : 167

Scopus : 447

Embase: 446

WOS: 244

Total : 1505 (with a minimum of 692 duplicates)
